# Transcriptomic and metabolic analysis unveils the mechanism behind leaf color development in *Disanthus cercidifolius* var. *longipes*

**DOI:** 10.3389/fmolb.2024.1343123

**Published:** 2024-02-06

**Authors:** Xiaoming Tian, Guangfeng Xiang, Hao Lv, Lu Zhu, Jing Peng, Gaofei Li, Cun Mou

**Affiliations:** Institute of Plant Conservation, Hunan Botanical Garden, Changsha, China

**Keywords:** *Disanthus cercidifolius* var. *longipes*, anthocyanin, carotenoid, leaf color, metabolomics

## Abstract

**Introduction:** Leaf coloration in *Disanthus cercidifolius* var. *longipes* results from the interplay of various pigments undergoing complex catalytic reactions.

**Methods:** We aimed to elucidate the mechanisms of pigment biosynthesis affecting leaf color transition in *D. cercidifolius* var. *longipes* by analyzing variations in pigment accumulation and levels of gene expression.

**Results:** We identified 468, 577, and 215 differential metabolites in green leaves (GL), gradual-color-changing leaves (GCCL), and red leaves (RL), respectively, with 94 metabolites shared across all comparisons. Metabolite accumulation patterns were similar among GL, GCCL, and RL, with flavonoids being the main differential metabolites. Delphinidin, malvidin, and petunidin derivatives were mostly accumulated in GCCL, whereas cyanidin, pelargonidin, and peonidin derivatives accumulated in RL. Transcriptome sequencing was used to identify differentially expressed genes. The expression of anthocyanin biosynthetic pathway genes was associated with anthocyanin accumulation patterns.

**Discussion:** Our findings reveal that the content of delphinidin, malvidin, petunidin, and carotenoids collectively determines the gradual transition of leaf color from green in spring and summer to green, purple, and orange-yellow in early autumn, whereas the content of cyanidin, peonidin, pelargonidin, and carotenoids together causes the autumnal transition to red or orange-red colors as leaves of *D. cercidifolius* var. *longipes* age.

## 1 Introduction

*Disanthus cercidifolius* var. *longipes* belongs to the genus *Disanthus*, a member of the Hamamelidaceae family (Yu et al., 2014). The *Disanthus* genus comprises only one species, *Disanthus cercidifolius*, which is native to the southern mountains of Japan. Of note, *D. cercidifolius* var. *longipes* is a variant of this species in the Sino-Japanese floristic region and a tertiary relict plant unique to China (Jiang et al., 2020). The color of the leaves of *D. cercidifolius* var. *longipes* gradually changes from green in early spring to red or orange-red in autumn, embellishing the lush evergreen broad-leaved forest. Owing to its flowers and autumn leaves, this plant has high ornamental and artistic value in gardening (Ulvrova et al., 2021).

Previous research on *D. cercidifolius* var. *longipes* has primarily focused on its floral characteristics, breeding system, flowering phenology, reproductive traits, population genetic diversity, genetic differentiation, and propagation techniques (Novikova et al., 2019; Meng et al., 2020). However, the molecular mechanisms driving the changes in leaf coloration in this nonmodel plant remain poorly understood. Typically, autumn leaf coloration is a complex process influenced by the type and concentration of pigments. Key pigments in plants, namely, chlorophylls (green), carotenoids (yellow to red), and anthocyanins (red to purple), collectively dictate the colors of plant tissues. Therefore, elucidating the biosynthesis of these primary pigments in both model and nonmodel plant species can improve understanding of the mechanisms underlying changes in leaf coloration.

In recent years, high-throughput sequencing technology has been increasingly used to explore the molecular mechanisms of metabolites in plants. Metabolomics is a branch of study that regards the organism as a dynamic whole and uses scientific data analysis techniques to investigate changes in metabolic profiles attributed to internal or external factors. This omics-based approach offers comprehensive insights into both static and dynamic changes within organisms (Li et al., 2004; Guo et al., 2023). For example, Li et al. (2021a) analyzed the metabolic and transcriptomic profiles of primary and secondary metabolites in purple leaf buds and green mature leaves of jujube. Their analysis of differentially accumulated metabolites showed that flavonoids were the major differential metabolites that determined leaf coloration. Similarly, Li et al. (2021b) explored the potential biosynthesis and regulatory mechanisms of anthocyanins in *Padus virginiana* L. The results showed that the differences in chlorophyll, carotenoid, and anthocyanin content correlated with the formation of leaf color in *Padus virginiana*. Sun et al. (2022) conducted metabolomic and transcriptomic analyses on the green, orange, and red leaves of *Acer triflorum* Komarov, identifying genes and metabolites associated with leaf color transitions. These studies suggested that the molecular mechanisms involved in the changes in leaf coloration are species-specific. Therefore, species-specific research into the molecular mechanisms of leaf color transitions is essential in guiding the genetic enhancement of these plants. It is necessary to study the mechanism of leaf color formation, analyze the chemical basis of leaf color and the mechanism of leaf color regulation to reasonably utilize the artistic value of *D. cercidifolius* var. *longipes*.

In this study, we performed metabolomic and transcriptomic analyses of *D. cercidifolius* var. *longipe*s leaves to investigate the mechanisms of biosynthesis of the main pigments in leaves across various developmental stages and to elucidate the relationships between genes involved in the biosynthesis and structure of coloring substances. Our findings provide new insights into the identification of functional genes and metabolites related to changes in leaf coloration. Furthermore, this research lays a theoretical foundation for leaf color breeding and superior variety selection in *D. cercidifolius* var. *longipe*s and other colored-leaf plants.

## 2 Results

### 2.1 Analysis of pigments and total flavonoids

**FIGURE 1:**
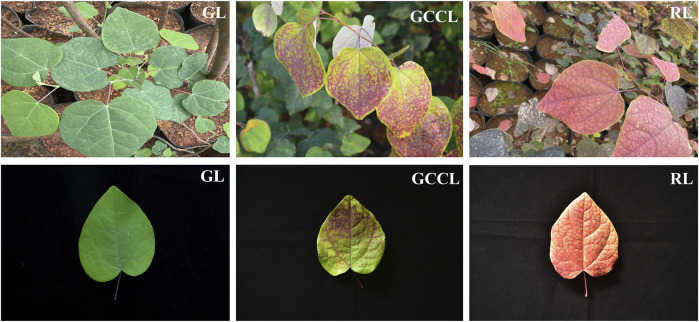
Changes in leaf color among samples of *Disanthus cercidifolius* var. *longipes* at different developmental stages. GL, green leaves; GCCL, gradual-color-changing leaves; RL, red leaves.

We observed the morphology and measured the content of pigments and total flavonoids in the leaves of *D. cercidifolius* var. *longipes* at various developmental stages. We detected notable differences in the color of green leaves (GL), gradual-color-changing leaves (GCCL), and red leaves (RL) (Figure 1). At different developmental stages (Table 1), the content of chlorophyll a and chlorophyll b in GL was significantly higher than that in GCCL and RL (*p* < 0.05). Our findings suggest that variations in the content of pigments contribute to the distinct colors observed in the leaves of *D. cercidifolius* var. *longipes*. In addition, we determined that the total flavonoid content in the leaves of *D. cercidifolius* var. *longipes* declined with increasing plant age.

**TABLE 1:** Changes in pigment and total flavonoid content of *Disanthus cercidifolius* var. *Longipes* leaves at different developmental stages.

Group name	Chlorophyll a content (mg/g)	Chlorophyll b content (mg/g)	Total chlorophyll content (mg/g)	Carotenoid content (mg/g)	Total anthocyanin content (μg/g)	Total flavonoid content (mg/g)
GL	1.86 ± 0.09 c	0.87 ± 0.03 b	2.74 ± 0.11 c	0.25 ± 0.01 a	14.11 ± 0.54 a	33.55 ± 1.48 c
GCCL	0.87 ± 0.03 b	0.60 ± 0.04 a	1.48 ± 0.07 b	0.32 ± 0.01 b	318.62 ± 5.42 b	28.93 ± 0.63 b
RL	0.61 ± 0.02 a	0.57 ± 0.02 a	1.19 ± 0.04 a	0.59 ± 0.03 c	473.02 ± 4.00 c	8.98 ± 0.36 a

Values are presented as the mean ± standard deviation (*n* = 3). Lowercase letters indicate significant difference as determined using the Tukey’s test (*p* < 0.05).

### 2.2 Multivariate analysis of the metabolome

**FIGURE 2:**
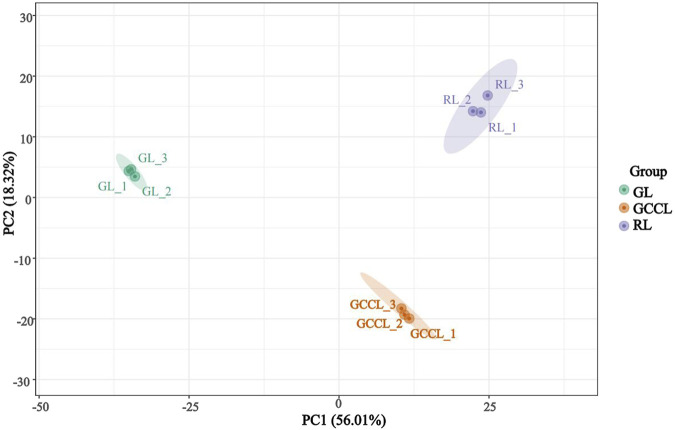
Principal Component Analysis (PCA) score plot of different colored leaves of *Disanthus cercidifolius* var. *longipes*.

Principal component analysis (PCA) revealed that several primary components accounted for the overall metabolic differences between groups and for the variability among samples within groups. We conducted multivariate analyses on the metabolic composition of GL, GCCL, and RL using Ultra High Performance Liquid Chromatography-Tandem Mass Spectrometry (UHPLC-MS/MS) data in both positive ion mode (POS) and negative ion mode (NEG). The PCA score plot showed a separation of raw data for different samples (Figure 2). We found that the two principal components, PC1 and PC2, explained 56.01% and 18.32% of the variance, respectively. In the PCA score plot, QC samples clustered together, indicating repeatability and reliability of the experiment. Furthermore, samples from GL, GCCL, and RL were distinctly separated in the plot, with repeats clustering together (Figure 2), demonstrating clear separation among the three groups.

The variable importance in projection (VIP) obtained from the orthogonal partial least squares-discriminant analysis (OPLS-DA) model (biological replicates ≥3) facilitated the preliminary screening of metabolites that differed between varieties or tissues. We further refined this list by considering the *p*-value/false discovery rate (FDR, biological replicates ≥2) from the univariate analysis. In this study, the criteria for screening of differential metabolites were as follows: (a) metabolites with a VIP >1, as the VIP value represents the impact level of intergroup differences of corresponding metabolites in classifying samples within the model, and metabolites with a VIP >1 are believed to be significantly different, and (b) metabolites that showed an intergroup difference with a fold change (FC) ≥2 or ≤0.5; these were considered significantly different. As shown in Table 2, we identified 468 differential metabolites between GL and GCCL, of which 253 were upregulated and 215 were downregulated. Between GL and RL, we identified 577 differential metabolites, of which 300 were upregulated and 277 downregulated. Between GCCL and RL, we identified 215 differential metabolites, of which 104 were upregulated and 111 downregulated.

**TABLE 2:** Statistics of differential metabolites in different colored leaves of *Disanthus cercidifolius* var. *longipes*.

Group name	All significantly different metabolites	Downregulated	Upregulated
GL vs. GCCL	468	215	253
GL vs. RL	577	277	300
GCCL vs. RL	215	111	104

**FIGURE 3:**
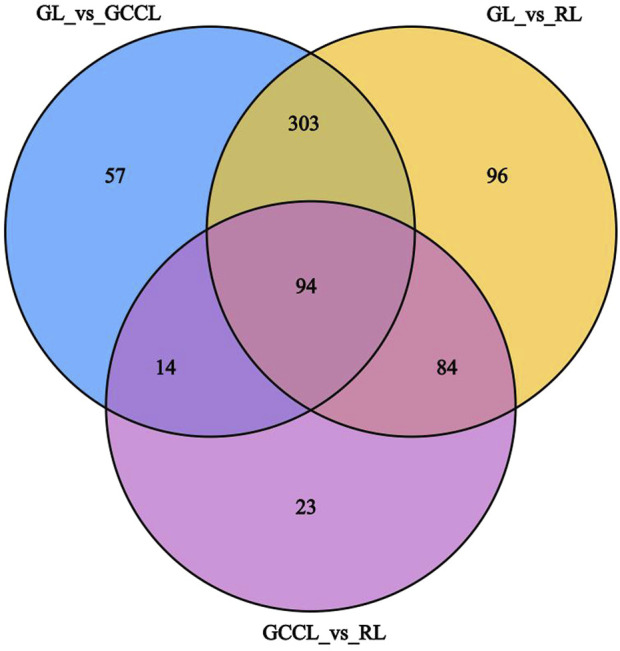
Venn diagram analysis of differential metabolites in GL, GCCL, and RL.

Venn diagram analysis of differential metabolites among differently colored leaves of *D. cercidifolius* var. *longipes* revealed 94 common differential metabolites across the three leaf groups (Figure 3). As shown in Table 3, the common differential metabolites (DCM) in GL, GCCL, and RL were categorized into 11 groups: flavonoids (29.79%), amino acids and derivatives (22.34%), lipids (18.09%), alkaloids (8.51%), phenolic acids (4.26%), organic acids (2.13%), nucleotides and derivatives (2.13%), quinones (2.13%), lignins and coumarins (1.06%), terpenoids (1.06%), and others (8.51%). Further analysis revealed that these differential metabolites were broadly classified into two groups. The first group comprised antioxidant components, including flavonoids, alkaloids, terpenes, quinones, phenolic acids, lignins, and coumarins. We observed that the concentration of most flavonoid differential metabolites was higher in GL compared with that in GCCL or RL. Conversely, we did not observe a consistent trend in the differential metabolites of alkaloids, terpenes, quinones, phenolic acids, lignins, and coumarins across GL, GCCL, and RL. The second group included amino acids and their derivatives, lipids, nucleotides and their derivatives, organic acids, and other compounds, with the concentration of most of these metabolites being significantly higher in GL than in GCCL and RL. In summary, our results indicate that GL, GCCL, and RL share similar metabolite accumulation patterns, with flavonoids being the primary differential metabolites during leaf color transitioning.

**TABLE 3:** Common upregulated (Up.) and downregulated (Down.) differential metabolites in different-colored leaves of *Disanthus cercidifolius* subsp. *Longipes*.

		GL vs. GCCL		GL vs. RL		GCCL vs. RL	
Component	DCM	Up	Down	Up	Down	Up	Down
Flavonoids	28	4	24	9	19	4	24
Amino acids and their derivatives	21	7	14	7	14	7	14
Phenolic acids	4	1	3	1	3	1	3
Nucleotides and their derivatives	2	0	2	0	2	0	2
Lignans and coumarins	1	0	1	0	1	0	1
Quinones	2	0	2	0	2	1	1
Other categories	8	3	5	3	5	4	4
Alkaloids	8	4	4	3	5	2	5
Terpenoids	1	1	0	1	0	1	0
Organic acids	2	0	2	0	2	0	2
Lipids	17	7	10	7	10	8	9
TOTAL	94	47	47	41	53	48	45

### 2.3 Analysis of polyphenols and anthocyanin-related differential metabolites

**FIGURE 4:**
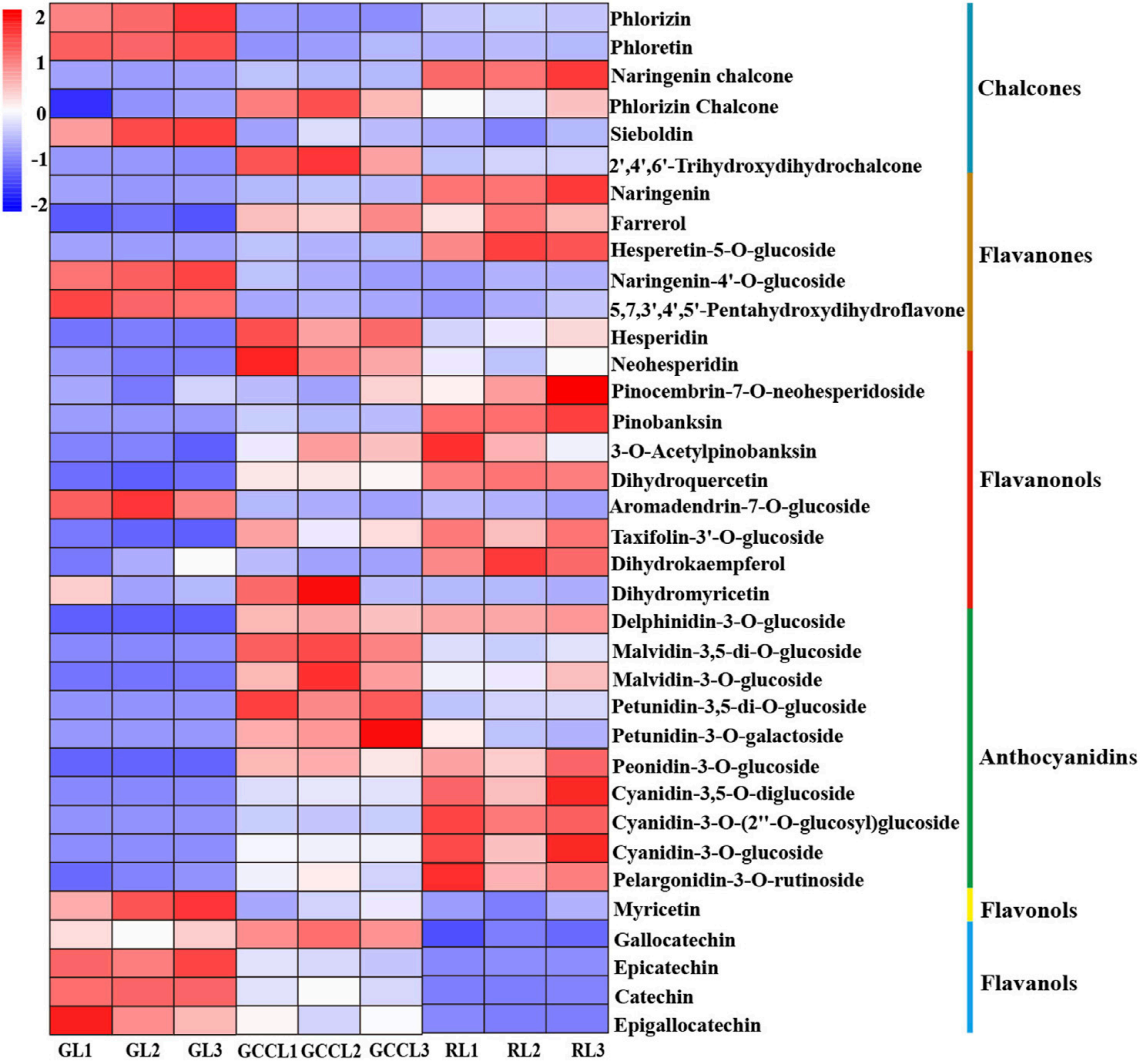
Heatmap of metabolites related to anthocyanin biosynthesis that were identified in different-colored leaves of *Disanthus cercidifolius* var. *longipes*. Note: each sample had three biological replicates, and each row represents a single metabolite. The abundance of each metabolite is shown by different colors. Red indicates high abundance, and blue indicates low abundance.

We also conducted a heatmap analysis on the integrated quantitative values of anthocyanin-related metabolites identified in the leaves of *D. cercidifolius* var. *longipes* (Figure 4). As shown in the figure, the accumulation of anthocyanins and their derivatives was significantly higher in RL compared with that in GL and GCCL. This finding suggested that these compounds are crucial in pigment formation in the leaves of *D. cercidifolius* var. *longipes*. In the initial stage of anthocyanin biosynthesis, phlordzin, phloretin, and 3-hydroxy-phlordzin are significantly accumulated in GL. However, we found that the accumulation of chalcone compounds such as naringenin chalcone, phlordzin chalcone, and 2′,4′,6′-trihydroxydihydrochalcone was higher in GCCL and RL. Flavanonols such as naringenin, dihydrokaempferol (DHK), dihydroquercetin (DHQ), and dihydromyricetin (DHM) were also significantly accumulated in RL. Notably, we observed that while RL showed the highest accumulation of naringenin among the three leaf color types, its DHM accumulation was the lowest. In the second stage of anthocyanin biosynthesis, we found that GL exhibited the highest content of myricetin, whereas other colorless flavonols, such as quercetin and kaempferol, were not detected in any of the three types of colored leaves. In the third stage of anthocyanin biosynthesis (Figure 4, third stage), we detected that the levels of accumulation of catechins, epicatechins, epigallocatechins, delphinidin derivatives, malvidin derivatives, petunidin derivatives, peonidin derivatives, cyanidin derivatives, and pelargonidin derivatives were higher in RL and GCCL than they were in GL. Among the three types of colored leaves, GCCL exhibited the highest epigallocatechin content, whereas GL had the lowest.

### 2.4 Statistical analysis of transcriptome data

We conducted sequencing on nine cDNA libraries constructed from the total RNA of GL, GCCL, and RL samples using the Illumina HiSeq 2000 platform to examine the sequence identity and abundance changes during leaf color transition. From the nine samples, we obtained 541,845,442 raw reads and 503,741,790 clean reads. We determined that the average Q20 and Q30 scores for the clean reads were 97.6% and 93.4%, respectively. The GC content varied between 0.433 and 0.448, with an average of 0.440. The annotation results of all unigenes are shown in (Table 4). Because no reference genome is available, gene functional annotation of the assembled unigenes was performed using the public databases. Using these databases, we functionally annotated 56,008 of the 95,712 (58.52%) assembled single genes.

**TABLE 4:** Number of annotated unigenes in *Disanthus cercidifolius* var. *Longipes* in various databases.

Database	Number of genes	Percentage (%)
KEGG	42342	44.24
Nr	55150	57.62
SwissProt	39699	41.48
TrEMBL	55022	57.49
KOG	32965	34.44
GO	47306	49.43
Pfam	36290	37.92

**FIGURE 5:**
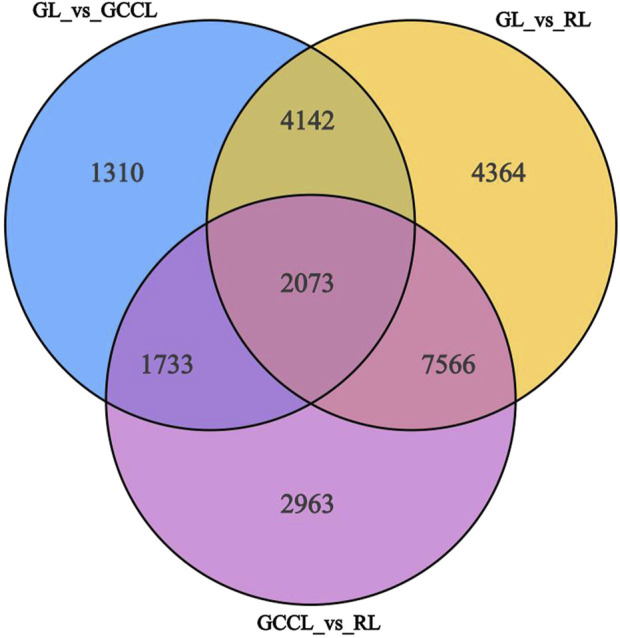
Venn diagram of DEGs among different-colored leaves of *Disanthus cercidifolius* var. *longipes*.

In transcriptome sequencing, we detected 24,152 nonredundant differentially expressed genes (DEGs). More specifically, we observed that in the GL vs. GCCL comparison, 4543 DEGs were upregulated and 4715 were downregulated; in the GL vs. RL comparison, 9585 DEGs were upregulated and 8560 were downregulated; and in the GCCL vs. RL comparison, 7837 DEGs were upregulated and 6498 were downregulated (Table 5; Figure 5).

**TABLE 5:** Number of differentially expressed genes (upregulated (Up.) and downregulated (Down.)) among different-colored leaves of *Disanthus cercidifolius* var. *Longipes*.

Group	Total	Down	Up
GL vs. GCCL	9258	4715	4543
GL vs. RL	18145	8560	9585
GCCL vs. RL	14335	6498	7837
GL vs. GCCL	9258	4715	4543

KEGG enrichment analysis revealed that in the GL vs. GCCL comparison, ko00941 (the flavonoid biosynthetic pathway), ko00940 (the phenylalanine biosynthetic pathway), and ko00942 (the anthocyanin biosynthetic pathway) were all significantly enriched (adjusted *p*-value ≤1). However, in the GL vs. RL comparison, only ko00941 (the flavonoid biosynthetic pathway) was significantly enriched (adjusted *p*-value ≤ 1). Finally, in the GCCL vs. RL comparison, both ko0090 (the carotenoid biosynthetic pathway) and ko00941 (the flavonoid biosynthetic pathway) were significantly enriched (adjusted *p*-value ≤ 1) (Table 6).

**TABLE 6:** Kyoto encyclopedia of genes and genomes pathway enrichment analysis of differentially expressed genes (DEGs; upregulated (Up.) and downregulated (Down.)) in different colored leaves of *Disanthus cercidifolius* var. *Longipes* (adjusted *p*-value ≤ 1).

	No.	Pathway ID	Pathway	Adjusted *p*-value	DEGs with pathway annotation	Up. DEGs	Down. DEGs
**GL vs. GCCL**	1	ko00196	Photosynthesis - antenna proteins	9.84176 × 10^−21^	39	0	39
	2	ko01110	Biosynthesis of secondary metabolites	1.7085 × 10^−19^	932	419	513
	3	ko00941	Flavonoid biosynthesis	5.59169 × 10^−11^	67	30	37
	4	ko01100	Metabolic pathways	1.95419 × 10^−10^	1501	636	865
	5	ko04626	Plant-pathogen interaction	9.93519 × 10^−06^	302	157	145
	6	ko00909	Sesquiterpenoid and triterpenoid biosynthesis	0.000302857	32	9	23
	7	ko00940	Phenylpropanoid biosynthesis	0.000410725	79	28	51
	8	ko00942	Anthocyanin biosynthesis	0.002229833	10	7	3
	9	ko00380	Tryptophan metabolism	0.009544345	70	27	43
	10	ko00592	Alpha-Linolenic acid metabolism	0.009772049	41	19	22
**GL vs. RL**	1	ko01110	Biosynthesis of secondary metabolites	1.86646 × 10^−16^	1631	735	896
	2	ko01100	Metabolic pathways	2.01987 × 10^−16^	2827	1288	1539
	3	ko00196	Photosynthesis - antenna proteins	1.15731 × 10^−15^	44	0	44
	4	ko00941	Flavonoid biosynthesis	1.55682 × 10^−06^	89	46	43
	5	ko00130	Ubiquinone and other terpenoid-quinone biosynthesis	0.000817108	67	36	31
	6	ko00195	Photosynthesis	0.00130987	70	7	63
	7	ko00073	Cutin, suberine, and wax biosynthesis	0.001440825	40	17	23
	8	ko04075	Plant hormone signal transduction	0.001763727	294	133	161
	9	ko00062	Fatty acid elongation	0.003968197	39	11	28
	10	ko00350	Tyrosine metabolism	0.005489975	58	30	28
	11	ko00710	Carbon fixation in photosynthetic organisms	0.006630647	94	21	73
	12	ko00402	Benzoxazinoid biosynthesis	0.00788083	26	10	16
	13	ko00860	Porphyrin metabolism	0.008782199	75	38	37
	14	ko00950	Isoquinoline alkaloid biosynthesis	0.009381599	46	23	23
**GCCL vs. RL**	1	ko01100	Metabolic pathways	2.91485 × 10^−14^	2188	1098	1090
	2	ko01110	Biosynthesis of secondary metabolites	7.69591 × 10^−14^	1267	608	659
	3	ko00196	Photosynthesis - antenna proteins	2.38062 × 10^−07^	31	1	30
	4	ko00062	Fatty acid elongation	0.000384745	35	11	24
	5	ko00380	Tryptophan metabolism	0.007865757	95	54	41
	6	ko00906	Carotenoid biosynthesis	0.009325175	73	44	29
	7	ko00941	Flavonoid biosynthesis	0.009708840	59	32	27

### 2.5 Analysis of DEGs in anthocyanin and carotenoid biosynthetic pathways

**FIGURE 6:**
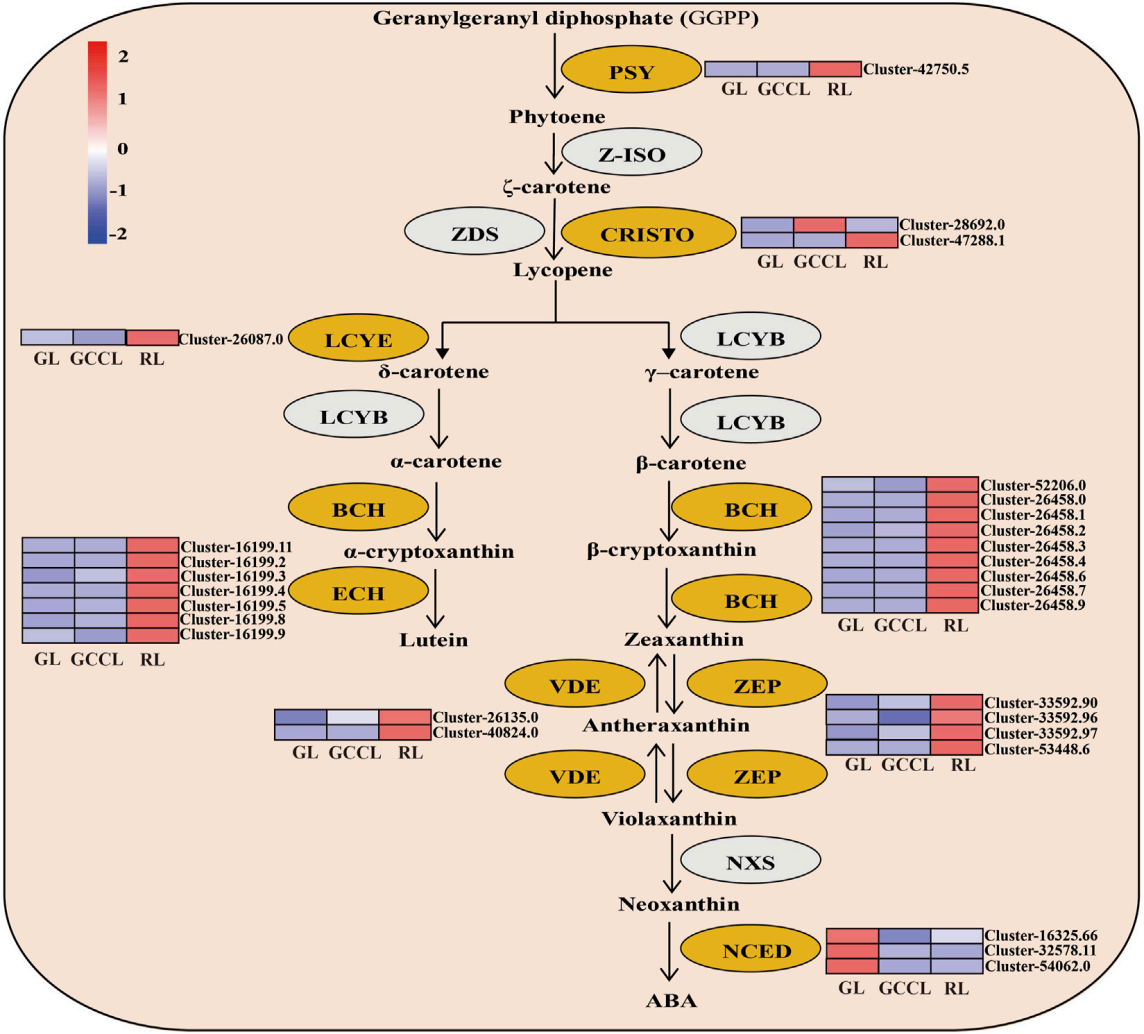
Heatmap of DEGs in the carotenoid biosynthetic pathway of *Disanthus cercidifolius* var. *longipes*. Note: DEGs were identified using limiting adjusted *p*-values < 0.05 and |log2 fold change (FC)| ≥1. Yellow ovals represent DEGs, and gray ovals indicate a difference in gene expression that was not significant. The color scale from minimum (blue) to maximum (red) indicates the levels of gene expression from low to high. In the heatmap, gene expression was calculated using Z-scores of Fragments Per Kilobase per Million (FPKM) mean values of three biological replicates. Cluster indicators on the right side of the heatmap denote the names of each gene.

A heatmap based on the fragments per kilobase of exon per million fragments mapped (Fragments Per Kilobase per Million, FPKM) values of DEGs related to carotenoids (Figure 6) showed that the expression of 82.75% of DEGs, including phytoene synthase (*PSY*), carotenoid isomerase (*CRISTO*), lycopene ε-cyclase (*LCYE*), β-ring hydroxylase (*BCH*), ε-ring hydroxylase (*ECH*), violaxanthin de-epoxidase (*VDE*), and zeaxanthin epoxidase (*ZEP*), was the highest in RL. We did not detect any significant differences in the expression of the abovementioned genes in GL and GCCL. The accumulation of carotenoids depends on the activity of their degradation enzymes. For example, 9-cis-epoxycarotenoid dioxygenase (*NCDE*) degrades carotenoids to form apocarotenoids, thereby reducing the accumulation of carotenoids in plants. As shown in Figure 6, expression of *NCDE* was the highest in GL, and it gradually decreased in GCCL and RL.

**FIGURE 7:**
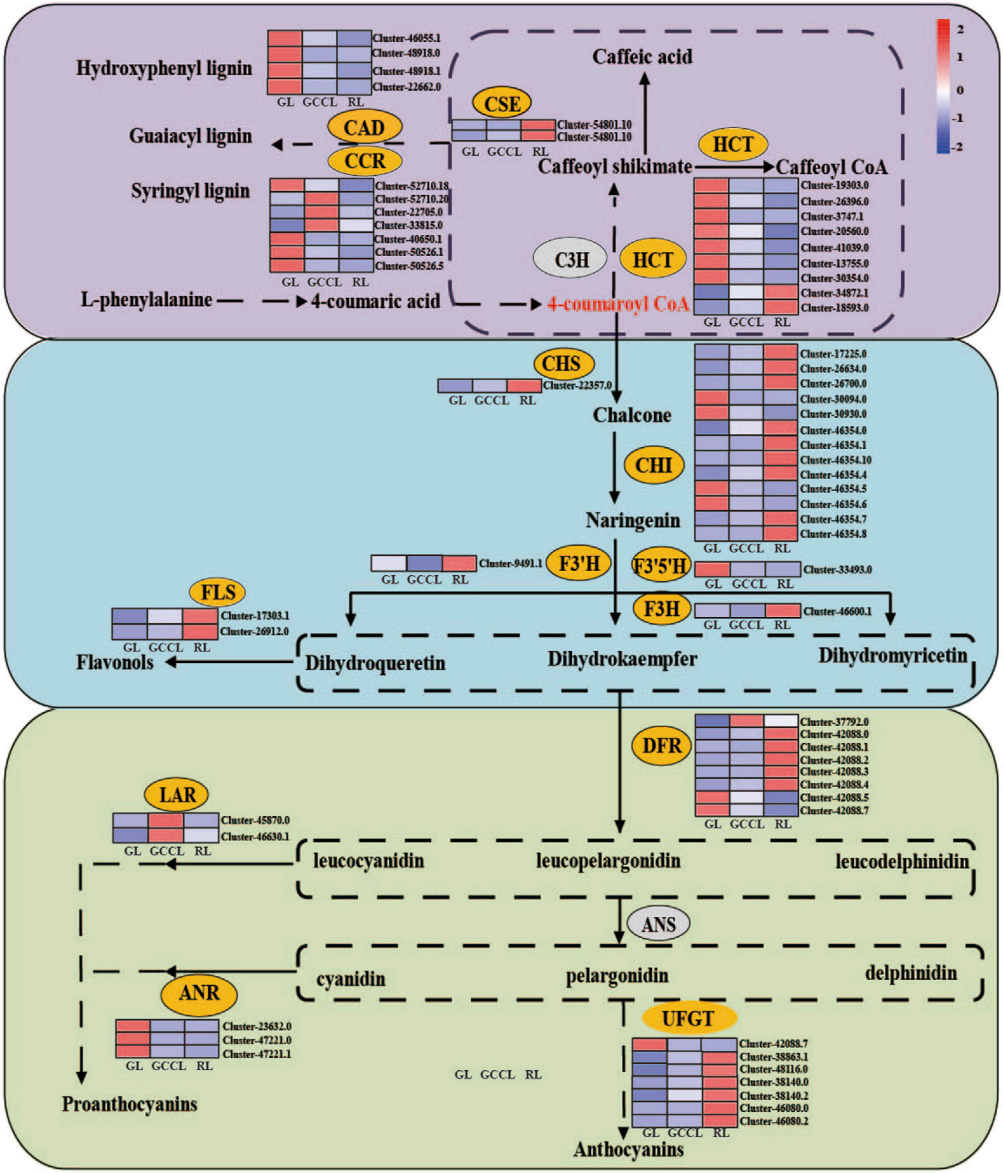
Heatmap of DEGs in the carotenoid biosynthetic pathway of *Disanthus cercidifolius* subsp. *longipes*. C3H, p-coumarate 3-hydroxylase; HCT, hydroxycinnamoyl-CoA shikimate/quinate hydroxycinnamoyl transferase; C3′H, p-coumaroyl shikimate 3′hydroxylase; CCR, cinnamoyl-CoA reductase; CAD, cinnamyl alcohol dehydrogenase; CSE, caffeoyl shikimate esterase; F5H, ferulate 5-hydroxylase; CHR, chalcone reductase; CHS, chalcone synthase; CHI, chalcone isomerase; IFS, isoflavone synthase; F3H, flavanone 3-hydroxylase; FNS, flavone synthase; F3′H, flavonoid 3′-hydroxylase; F3′5′H, flavonoid 3′5′-hydroxylase; DFR, dihydroflavonol 4-reductase; FLS, flavonol synthase; LAR, leucoanthocyanidin reductase; ANS, anthocyanin synthase; ANR, anthocyanidin reductase; UFGT, UDP-glucose flavonoid glycosyltransferase.

Anthocyanins are produced from phenylalanine through the flavonoid biosynthetic pathway. Following glycosylation, methylation, and acetylation, anthocyanin monomers are transformed into variously colored anthocyanins. We conducted a heatmap analysis (Figure 7) based on the FPKM values of DEGs associated with the anthocyanin synthesis pathway. We found that DEGs in the phenylpropanoid metabolic pathway included hydroxycinnamoyl-CoA shikimate (*HCT*), cinnamyl alcohol dehydrogenase (*CAD*), cinnamoyl-CoA reductase (*CCR*), and caffeoyl shikimate esterase (*CSE*). Among these genes, 59.09% exhibited the highest expression in RL, 13.04% in GCCL, and 26.08% in RL.

We observed that in the first stage of anthocyanin biosynthesis (the flavonoid biosynthetic pathway), chalcone synthase (*CHS*), chalcone isomerase (*CHI*), flavanone 3-hydroxylase (*F3H*), flavonoid 3′-hydroxylase (*F3′H*), flavonoid 3′5′-hydroxylase (*F3′5′H*), and flavonol synthase (*FLS*) were among the identified DEGs. Of these genes, 36.84% were upregulated in GL, whereas 57.89% were upregulated in RL. We noticed that the expression of *F3′H* was higher in GCCL compared with that in both RL and GL. We identified 13 DEGs among *CHI* genes, and the expression of four of them was the highest in GL, whereas nine showed maximum expression in RL. In addition, we found that the expression levels of *CHS*, *FLS*, and *F3H* were higher in RL than in both GL and GCCL. Conversely, *F3′5′H* was more highly expressed in GL than in RL and GCCL.

In the second stage of anthocyanin biosynthesis, we identified eight DEGs among *DFR* genes from the three types of leaf coloration. Of these, the expression of one was the highest in GCCL, whereas five and two *DFRs* showed maximum expression in RL and GL, respectively. We also determined that the expression of all anthocyanidin reductase (*ANR*) genes was the highest in GL, whereas leucoanthocyanidin reductase (*LAR*) showed the highest expression in GCCL and lower expression in GL and RL.

Due to the rarity of free form anthocyanins under natural conditions, anthocyanins typically remain stable in glycosylation form. UDP glucose flavonoid glycosyltransferase (UFGT) is the GT1 family of the glycosyltransferase superfamily. UFGT could be classified into three different groups-UF3GT, UF5GT, and UF7GT-based on the regioselectivity of flavonoid glycosylation (Li et al., 2016). Yao et al. (2019) found that the biological functions of UFGTs in plants are complex and diverse, and the combination of phylogenetic analysis and experimental analysis is the most effective way to identify UFGTs. In the third stage of anthocyanin biosynthesis, we identified 7 DEGs in the UFGTs. According to phylogenetic tree analysis (Supplementary Figure S1), Cluster42088.7, Cluster46080.0, and Cluster46080.2 were located in the UF5GT protein branch, Cluster38140.0 and Cluster38140.2 were located in the UF7GT protein branch, and Cluster38863.1 and Cluster48116.0 were located in the UF3GT protein branch. The expression of Cluster 42088.7 was lower in RL, while the expression of other genes is higher in GL and GCCL.

### 2.6 Quantitative real-time PCR (qRT-PCR) validation of DEGs

**FIGURE 8:**
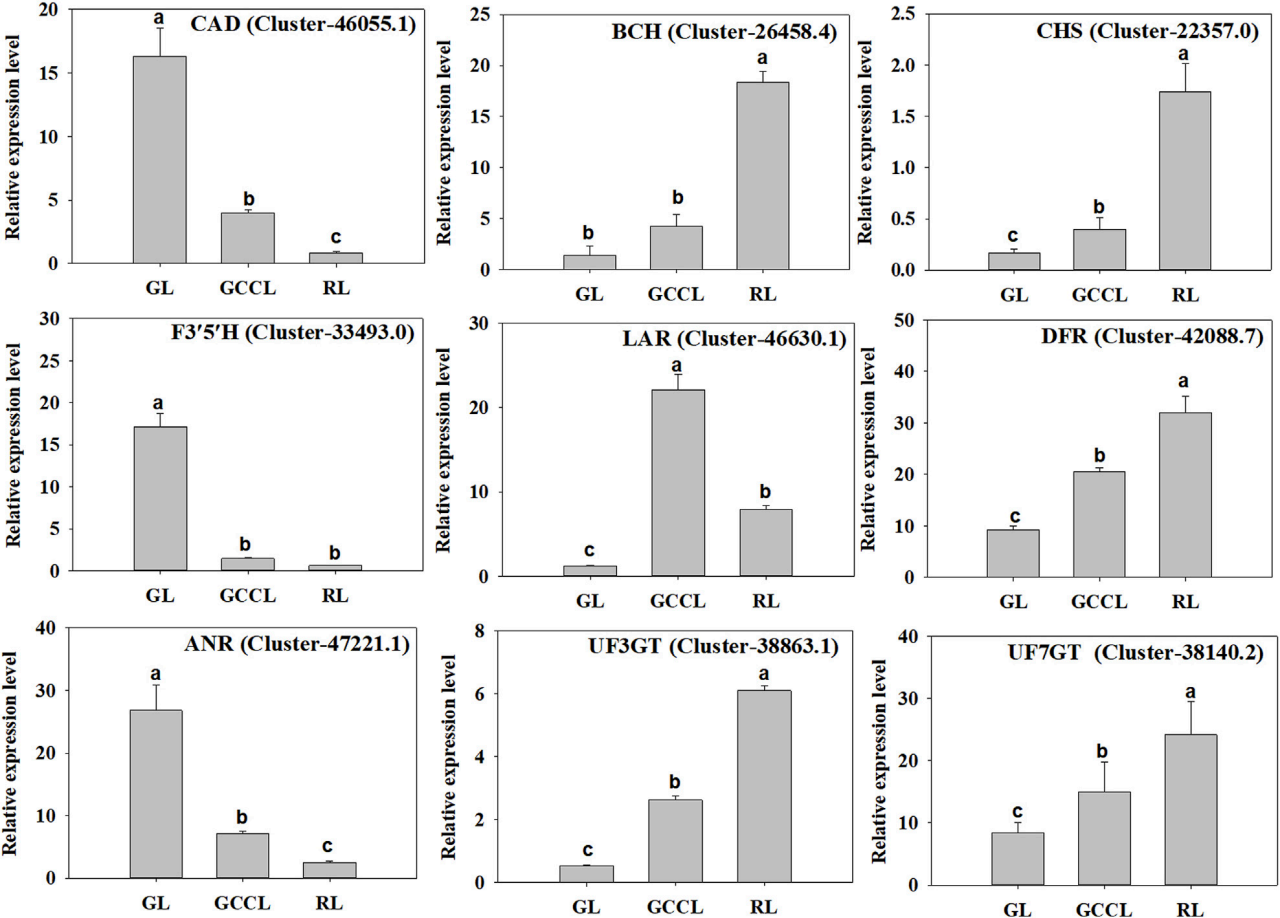
qRT-PCR validation of the levels of expression of nine DEGs identified using RNA-seq. Note: the *y*-axis indicates the relative level of gene expression analyzed using qRT-PCR (2^−ΔΔCq^). The *x*-axis indicates different leaf samples. Values are presented as means ± SD of three independent measurements. Bars with different lowercase letters indicate significant differences (*p* < 0.05).

To validate the transcriptome data, we selected nine DEGs related to carotenoid and anthocyanin biosynthesis (six upregulated and three downregulated) in GL, GCCL, and RL for qRT-PCR analysis. We found that the expression patterns of these candidate genes were consistent with those obtained from RNA-seq analysis, confirming the consistency between the Illumina sequencing and qRT-PCR data (Figure 8).

## 3 Discussion

The color of plant leaves is determined by the pigments in the vacuoles of leaf cells, especially by the levels and ratios of chlorophyll, carotenoids, and anthocyanins (Borges et al., 2022). Among these, carotenoids primarily contribute to the formation of yellow, orange, and red colors, whereas anthocyanins mainly result in red, pink, purple, and blue leaves (Clara, 2022). In this study, we explored the variation in pigment accumulation and levels of gene expression at different developmental stages of *D. cercidifolius* var. *longipes*.

The carotenoid metabolic pathway mainly includes carotenoid synthesis and degradation (Wang et al., 2023). First, GGPP is converted to 15-cis-phytoene by PSY, which is subsequently transformed into lycopene through the actions of PDS, ZDS, and CRTISO. From lycopene, the pathway then diverges into two branches: in one, lycopene is sequentially catalyzed by LCYE and LCYB to form α-carotene, which is ultimately converted to lutein by BCH and ECH, and in the other, lycopene undergoes dual catalysis by LCYB to produce β-carotene, which is then sequentially transformed into antheraxanthin, violaxanthin, and neoxanthin under the action of ZEP and NXS (Diao et al., 2023). Additionally, VDE reversibly regulates these three xanthophylls. The carotenoid degradation pathway is mediated by NCED, which decomposes carotenoids to form apocarotenoids (Koschmieder et al., 2022). In this study, we found that during leaf color transition, the expression levels of PSY, CRISTO, LCYE, BCH, ECH, VDE, and ZEP exhibited an upward trend, leading to a progressively increased accumulation of their respective products. Consequently, the leaves underwent a gradient color shift from yellow to yellow-red or orange-red. The expression level of NCED showed a negative correlation with that of carotenoids, consistent with the findings of the study by Shen et al. (2014), where silencing of PacNCED1 inhibited the red coloring of sweet cherry cv. Hong Deng fruits.

Based on functionality, the anthocyanin biosynthetic pathway can be divided into early and late biosynthetic enzymes, specifically those involved in the synthesis of flavonoids, anthocyanins, catechins, and flavonols (Khaleghnezhad et al., 2019). Phenylalanine is converted to 4-coumaroyl-CoA via PAL, C4H, and 4CL (Ma et al., 2022). This compound is then converted by CHS, CHI, F3H, F3′H, and F3′5′H to the direct precursors of anthocyanin synthesis. Through DFR, colorless anthocyanins are formed and further catalyzed by ANS/LDOX to produce various colored anthocyanins; finally, UFGT catalyzes these molecules into stable anthocyanins (Bao et al., 2022; Zhao et al., 2023).

Two important branches are present downstream of the phenylpropanoid metabolic pathway: the lignin synthesis pathway and the flavonoid synthesis pathway. Briefly, 4-coumaroyl-CoA enters the specific pathway for lignin synthesis through HCT and is converted to monolignols such as coumaryl alcohol, coniferyl alcohol, and sinapyl alcohol by CAD, CCR, and CSE, respectively. According to previous research, the key genes implicated in anthocyanin synthesis, *PAL*, *C4H*, and *4CL*, which promote the synthesis and transport of anthocyanins are upregulated (Ming et al., 2021). Conversely, the upregulation of genes (including *HCT*, *CAD*, and *CCR*) causes the crucial intermediate 4-coumaroyl-CoA to be primarily directed toward the lignin synthesis pathway instead of the flavonoid synthesis pathway. This redirection decreases the accumulation of anthocyanins. Our findings indicate that the majority of DEGs in the phenylpropanoid metabolic pathway among the three types of differently colored leaves of *D. cercidifolius* var. *Longipes* exhibited higher expression in GL, aligning with the lower total anthocyanin content in GL. These results suggest that during the leaf color transition of *D. cercidifolius* var. *Longipes*, the accumulation of anthocyanins in the leaves is regulated by the limiting of the amount of intermediates channeled into the anthocyanin synthesis pathway.

In the first stage of anthocyanin synthesis, the expression of CHS is positively correlated with anthocyanin synthesis (Wong et al., 2002). Our findings indicated that naringenin chalcone and naringenin accumulated the most in RL, with the expression levels of *CHS*, *F3H*, *F3′H*, *FLS*, and most *CHI* genes also being the highest in RL. Therefore, DHK and DHQ accumulation was greater in RL. However, DHM accumulation was the highest in GCCL, followed by that in GL and RL leaves. This was contradictory to the observation that GL expressed the highest level of *F3′5′H*. A potential explanation is that many *F3′5′H* genes may catalyze reactions in other flavonoid synthesis pathways aside from that of anthocyanin synthesis, leading to a discrepancy between metabolite accumulation and gene expression. FLS catalyzes the conversion of flavanonol into three different flavonols (quercetin, kaempferol, and myricetin) (Luo et al., 2015). In petunias, FLS shifts the ratio of flavonols to anthocyanins, resulting in changes in the color of the flowers (Luo et al., 2015). A study by Luo et al. (2014) found strong competition between *DFR* and *FLS* to act on DHM in *Muscari armeniacum*. We observed that during the leaf color transition of *D. cercidifolius* var. *Longipes*, the expression of *FLS* was gradually increased. As a result, RL should have exhibited a higher content of quercetin, kaempferol, and myricetin. However, our study revealed that the myricetin content was the highest in GL, followed by that in GCCL and RL leaves, whereas no other colorless flavonols (quercetin, kaempferol, etc.) were detected in the three types of differently colored leaves. This might be attributed to GCCL and GL producing more DHM than RL did in the initial stage.

In the second stage of anthocyanin synthesis, DFR catalyzes the conversion of three flavanonols (DHK, DHQ, and DHM) to their corresponding colorless proanthocyanidins (propelargonidins, procyanidins, and prodelphinidins). The substrate specificity of DFR is crucial for determining the compounds produced. In many plants, DFR efficiently recognizes DHK, DHQ, and DHM. In this study, we found that the expression patterns of the *DFR* gene varied among the three types of differently colored leaves. This variation may arise from the inconsistent expression patterns of *F3′H*, *F3H*, and *F3′5′H* in the first stage, leading to different accumulations of the three flavanonols. In addition, we found that eight *DFR* genes were involved in the efficient recognition and catalysis of the three flavanonols in the three types of different-colored leaves.

In the third stage of anthocyanin synthesis, ANS catalyzes the conversion of colorless proanthocyanidins to colored anthocyanin precursors (Luo et al., 2015), whereas LAR catalyzes the conversion of proanthocyanidins to the respective flavonoids. We found that the expression level of the *LAR* gene in GCCL was higher than that in both GL and RL. However, no significant differences were detected in the expression of *ANS* among the three types of different colored leaves, indicating that the unstable, colorless anthocyanidins primarily entered the synthesis pathway for proanthocyanins over anthocyanins. Furthermore, ANR catalyzes the reduction of anthocyanidin, leading to the synthesis of epicatechin, epiafzelechin, and epigallocatechin. Xia and Gao (2014) reported that the upregulation of the expression of *LAR* and *ANR* led to an increase in the accumulation of proanthocyanidins (a product of catechins combined with epicatechins) in tea leaves. In our study, the expression of *ANS* was higher in GL compared with that in both GCCL and RL, and the accumulation of catechins, epicatechins, and epigallocatechins in GL and GCCL exceeded that in RL, further confirming the previous results. However, afzelechin and epiafzelechin were not detected in our study, which could be attributed to the disruption in the LAR-catalyzed production of colorless pelargonidin and ANR-catalyzed production of pelargonidin. The unstable, colorless anthocyanidins are further converted into stable anthocyanidins by glucosyltransferase (GT). Of note, *GT* has been shown to exhibit a strong specificity for spatiotemporal expression and strong substrate specificity. The biological functions of *UFGT*s in plants are complex and diverse, which making it difficult to accurately determine their function and specificity based on their protein sequences (Yao et al., 2019), accordingly, the expression patterns of *UFGT* varied among the three types of differently colored leaves in this study.

In summary, by analyzing the expression patterns of genes related to the anthocyanin biosynthetic pathway, we found that the expression levels of *CCR*, *HCT*, *CHI*, *F3′5′H*, and *DFR* did not exhibit specific patterns. Conversely, the expression levels of *CAD*, *CSE*, *CHS*, *F3′H*, *F3H*, *FLS*, *LAR* significantly affected and were thus consistent with the accumulation pattern of anthocyanidins. Regarding genes associated with the carotenoid biosynthetic pathway, the expression levels of *PSY*, *CRISTO*, *LCYE*, *BCH*, *ECH*, *VDE*, and *ZEP* showed an increasing trend, whereas the expression level of *NCDE* gradually decreased. These trends were consistent with the changes in carotenoid content observed in the three types of differently colored leaves, suggesting a regulatory role of these genes in carotenoid synthesis.

Anthocyanin-related compounds in living plants predominantly occur in the form of anthocyanins, originating from anthocyanidins (Huang et al., 2020). Over 600 anthocyanins have been isolated and identified in nature, primarily derived from six anthocyanidins: cyanidin (50%), delphinidin (12%), pelargonidin (12%), peonidin (12%), petunidin (7%), and malvidin (7%) (Chen et al., 2022). Specifically, peonidin, petunidin, and malvidin are derived from cyanidin and delphinidin. In this study, derivatives of all six of these anthocyanins were detected. The accumulation of delphinidin (1), malvidin (2), and petunidin derivatives (2) was the highest in GCCL, whereas that of cyanidin (3), pelargonidin (1), and peonidin derivatives (1) was the highest in RL. During the gradual color transition of *D. cercidifolius* var. *Longipes* leaves from green to red, the content of blue-violet delphinidin derivatives, malvidin derivatives, and petunidin derivatives, as well as that of yellow carotenoids, was significantly increased, with the highest content observed in GCCL. This resulted in a gradual transition of leaf color from green to purple and eventually to orange-yellow. Therefore, we inferred that early-stage leaf color changes in *D. cercidifolius* var. *Longipes* are determined by the content of delphinidin, malvidin, petunidin, and carotenoids. As the leaves aged, the concentration of brick-red or magenta cyanidin derivatives, peonidin derivatives, and red or orange pelargonidin derivatives substantially increased, exhibiting the highest levels in RL. The content of carotenoids was also significantly increased, with the rate of increase being three-fold of that observed during the GL to GCCL transition. Consequently, the leaves became red or orange-red. From this, we inferred that the color of aging *D. cercidifolius* var. *Longipes* leaves is primarily determined by the content of cyanidin, peonidin, pelargonidin, and carotenoids.

## 4 Materials and methods

### 4.1 Materials and sampling

The experimental materials were leaf samples of *D. cercidifolius* var. *Longipes* sourced from the nursery at Hunan Botanical Garden (113° E, 28°20′N; Changsha, Hunan, China). In 2022, fresh leaves growing normally under optimal outdoor lighting conditions and showing no signs of pest infestation were selected based on their growth and developmental stages. Leaves were sampled at three developmental stages. On 25 July 2022, GL samples, representing mature leaves with green coloration, were collected. On 25 September 2022, GCCL samples, displaying a gradient coloration of green, purple, and orange-yellow and indicating the onset of leaf senescence, were collected. On 25 November 2022, RL samples, displaying red or orange-red coloring and representing fully senescent leaves entering the defoliation period, were collected. Following collection, the leaves were promptly transported to the laboratory, flash-frozen in liquid nitrogen, and stored at −80 °C for subsequent use. Three biological replicates were included for each leaf sample.

### 4.2 Measurement of the contents of pigments and total flavonoids

Chlorophyll and carotenoid content was determined as previously described (Tunç et al., 2023). Briefly, 0.2 g of *D. cercidifolius* var. *Longipes* leaves were finely cut and placed in a 50 mL centrifuge tube, followed by the addition of 45 mL of an acetone:anhydrous alcohol (1:1) mixture. The tube was incubated in the dark for 24 h. Absorbance was measured at wavelengths of 646, 663, and 470 nm. The chlorophyll-a, chlorophyll-b, and carotenoid (Car) content was calculated according to the following equations:

$$\text{Chlorophyll}-a=\left(12.21\;\times A_{663}-2.81\times A_{645}\right)\times V/1000\times W$$ (1)

$$\text{Chlorophyll}-b=\left(20.13\;\times A_{645}-5.03\times A_{663}\right)\times V/1000\times W$$ (2)

$$\text{Total chlorophyll}=(\left(12\;\times A_{663}\right)\;-\;\left.\left(3.11\;\times A_{645}\right)\right)\times V/1000\times W$$ (3)

and

$$\text{Car}=\left(\left(1000\;\times A_{480}\right)\;-\;\left(1.12\;\times \text{ Chla }\right)-\left(34.7\;\times \text{ Chlg}\right)\right)/\;245\times V/1000\times W$$ (4)

where A is the absorbance, V is the volume (mL), and W is the fresh weight of leaves (g). Three technical replicates were performed for each measurement.

Total anthocyanin content was determined as previously described (Khan and Akhtar, 2012). Briefly, 0.1 g of *D. cercidifolius* var. *Longipes* leaves was ground in liquid nitrogen and mixed with 0.5 mL of extraction solution. The mixture underwent ultrasonic extraction for 30 min and was then centrifuged at 8000 *g* at 25 °C for 10 min. The supernatant was collected, and 200 μL of the sample was transferred to a 96-well plate. Absorbance values at 530, 620, and 650 nm were recorded. Anthocyanin content (µg/g) was calculated according to the following formula:

$$\text{anthocyanin content }\left(µg/g\right)=\left[\Delta A\times V/\left(\varepsilon \times d\right)\times M\times F\times 10^{6}\right]/W=33.4\times \Delta A\times F/W$$ (5)

where ΔA is the absorbance of total anthocyanins and is equal to (A_530_−A_620_) −0.1 (A_650_−A_620_), V is the volume of the extract (1 × 10^−3^ L), ε is the molar extinction coefficient of total anthocyanins (2.69 × 10^4^ L/mol/cm), d is the diameter of the 96-well plate (0.5 cm), M is the relative molecular weight of total anthocyanins (449.2 g/mol), F is the dilution ratio, and W is the sample weight (g).

Total flavonoid content was determined as previously described (Kegode et al., 2023). Briefly, 2 g of *D. cercidifolius* var. *Longipes* leaves was ground in liquid nitrogen and then placed in a 95% ethanol solution (30 mL). The mixture was subjected to ultrasonic extraction in a water bath at 60 °C for 30 min, and this step was repeated three times. The mixture was then filtered through a filter paper, and the filtrate was dried using a rotary evaporator. The residue was resuspended in methanol (50 mL) and then filtered through a 0.45 μM membrane (Millipore, USA). Next, 5 mL of the extract was combined with 5% NaNO_2_ solution (0.3 mL) and allowed to stand for 6 min. Subsequently, 5% Al(NO_3_)_3_ solution (0.3 mL) was added, and the mixture was left for an additional 6 min before adding 4% NaOH solution (4.4 mL). After a 6 min incubation, the absorbance was measured at 510 nm, with rutin used as a standard for the calibration curve.

### 4.3 Metabolite extraction

Sample preparation, extract analysis, metabolite identification and quantification were performed at Wuhan Metware Biotechnology Co., Ltd. (Wuhan, China). For metabolite extraction, leaf samples were quickly freeze-dried and then ground with zirconia beads for 1.5 min at 30 Hz using a mixer mill (MM 400, Retsch). Fifty milligram of the resulting powder was extracted with 0.5 mL of a methanol/water/hydrochloric acid solution (799:200:1, V/V/V). The mixture was vortexed for 10 min and subsequently ultrasonicated for 10 min. After centrifugation at 12,000 × *g* for 3 min at 4 °C, the supernatant was collected and filtered through a 0.22 μm polytetrafluoroethylene membrane (ANPEL, Shanghai, China).

The UHPLC-MS/MS analysis was performed following the method described by Ye et al. (2022). The leaf sample extracts were analyzed using an UHPLC-MS/MS system (UHPLC, ExionLC™ AD, https://sciex.com.cn/; MS, Applied Biosystems 4500 QTRAP, www.appliedbiosystems.com.cn/), equipped with an ESI Turbo Ion-Spray interface. The UHPLC conditions were as follows: column, Agilent SB-C18 (1.8 µm, 2.1 mm × 100 mm); mobile phase, solvent A (pure water with 0.1% formic acid) and solvent B (acetonitrile with 0.1% formic acid). The gradient program used for separation was as follows: 0.00 min, 5% B; 5%–95% B over 9.00 min; 10.00–11.10 min, 95%–5% B over 14 min. The flow rate of solvent was set at 0.35 mL/min and the column oven was set to 40 °C. The sample injection volume was 4 μL. The ESI source operation parameters were as follows: source temperature, 550 °C; ion spray voltage (IS), 5500 V (positive ion mode)/−4500 V (negative ion mode); ion source gas I (GSI), gas II (GSII), curtain gas (CUR) were set at 50, 60, and 25.0 psi, respectively; the cinnamyl alcohol dehydrogenase (CAD) was high. Instrument tuning and mass calibration were performed with 10 and 100 μmol/L polypropylene glycol solutions in triple quadrupole (QQQ) and linear ion trap (LIT) modes, respectively. QQQ scans were acquired as multiple reaction monitoring (MRM) experiments with collision gas (nitrogen) set to medium. Declustering potential (DP) and collision energy (CE) for individual MRM transitions were optimized. A specific set of MRM transitions was monitored for each period according to the metabolites eluted within this period (Ye et al., 2022).

### 4.4 Metabolome analysis and data processing with UHPLC-MS/MS

Metabolites were identified by comparing the mass/charge (m/z) values, retention time, and fragmentation patterns with the standards hosted on the database curated by Metware Biotechnology Co., Ltd. Metabolite data were log2-transformed for statistical analysis to improve normality and were normalized. Normalized metabolite data from nine leaf samples of three different colors were analyzed to compare the metabolite composition. Hierarchical cluster analysis (HCA) and orthogonal partial least squares discriminant analysis (OPLS-DA) were conducted on these samples using the R software to study metabolite accession-specific accumulation. The *p* and fold change values were set to 1 and 2.0, respectively. Differentially accumulated metabolites (DAMs) with variable importance in the project VIP values (VIP ≥1) and fold change ≥2 or ≤0.5 were considered as significantly changed metabolites. Venn diagrams were used to illustrate the number of DAMs. The Kyoto encyclopedia of genes and genomes (KEGG) database with a *p*-value < 0.01 was used to study DAMs.

### 4.5 RNA sequencing and functional annotation

Briefly, 0.1 g of leaves was used for RNA extraction. The quantity, purity, and integrity of the total RNA were assessed using the Qubit 2.0 Fluorometer (Life Technologies, Carlsbad, CA, USA), NanoPhotometer^®^ spectrophotometer (IMPLEN, Westlake Village, CA, US), and Agilent 2100 Bioanalyzer (Agilent Technologies, Santa Clara, CA, USA), respectively. PolyA mRNA was isolated from total RNA using oligonucleotide-coated magnetic beads. RNA was fragmented into short segments using a fragmentation buffer, and first-strand cDNA was synthesized using a reverse transcriptase. The PCR amplification of cDNA was used for the construction of nine libraries, comprising the three samples, which was used for subsequent RNA-seq analysis.

After amplification and purification, cDNA libraries were prepared and sequenced using the Illumina Novaseq 6000 system (Chen et al., 2021). The raw reads were transformed from the sequential raw image data using CASAVA base recognition. Sequencing data were filtered using the fastp software (version 0.20.0) (Chen, 2023) by removing low-quality reads and adapters to obtain high-quality clean sequences. The clean data from the nine libraries were then used for RNA *de novo* assembly with Trinity version 2.06 (Grabherr et al., 2011). All the assembled transcripts were queried against the NCBI non-redundant (Nr) database (ftp://ftp.ncbi.nih.gov/blast/db/), Swiss-Prot protein database and Trembl (http://www.uniprot.org/), eukaryotic ortholog groups (KOG) database (version 1.0), gene ontology (GO) database (https://www.geneontology.org), and Kyoto encyclopedia of genes and genomes (KEGG) pathway database (http://www.genome.jp/kegg) using BLASTX to identify the proteins with the highest sequence similarity to the given scripts for retrieving their function annotations. We then used the HMMER software for comparison with the Pfam database (version 33.0) (http://pfam.xfam.org/) to obtain annotation information of Unigene. Fragments per kilobase million reads (FPKM) values were used to calculate gene expression levels using feature counts. The gene expression levels were estimated using RSEM (version 1.2.26) (Li et al., 2011). The RNA-seq data have been deposited in the NCBI Sequence Read Archive (NCBIvSRA) under accession number PRJNA1051698. The BioSample accession number is SAMN38771237.

### 4.6 Analysis of DEGs and KEGG enrichment

To estimate the differential expression of genes, the count matrix of different comparison groups was calculated using the R package DESeq2 (1.20.0). The obtained *p*-values were adjusted using the Benjamini and Hochberg’s FDR. Genes with an adjusted *p*-value < 0.05 and |log2 FC| >1 were considered DEGs. The KOBAS 2.0 software was used to test the statistical enrichment of DEGs in KEGG pathways.

### 4.7 Validation using qRT-PCR

To validate the transcriptome data, the relative expression of nine DEGs identified in the transcriptome analysis was assessed by performing qRT-PCR, using three biological and three technical replicates. We performed RNA extraction and cDNA synthesis using the TIANGEN Total RNA Extraction Kit (TIANGEN, Beijing, China) and PrimeScript RT Reagent Kit with gDNA Eraser (TaKaRa, Kyoto, Japan), respectively. The specific primers for selected DEGs were designed using the Primer premier 5.0 software (Premier Biosoft, Palo Alto, CA, USA) and are shown in (Supplementary Table S1). The *actin* gene was used as an internal control. qRT-PCR detection was performed on the ABI 7500 Fast Real-Time PCR System using the TaKaRa SYBR Green Master Mix Kit (TaKaRa, Beijing, China).

## 5 Conclusion

In this study, we found that leaf coloration in *D. cercidifolius* var. *Longipes* results from the interplay of various pigments undergoing a series of complex catalytic reactions. The coordinated upregulation of anthocyanin biosynthesis-related genes is the main reason for the increase in the content of anthocyanidins. In addition, the content of delphinidin, malvidin, petunidin, and carotenoids collectively determines the gradual leaf color transition from green in spring and summer to green, purple, and orange-yellow in early autumn, whereas the content of cyanidin, peonidin, pelargonidin, and carotenoids together causes the autumnal transition to red or orange-red colors as leaves of *D. cercidifolius* var. *Longipes* age.

## Data Availability

The RNA-seq data have been deposited in the NCBI Sequence Read Archive (NCBIvSRA) under accession number PRJNA1051698. The BioSample accession number is SAMN38771237.
