# Supplementary figures and images for: Transcriptomic and metabolic analysis unveils the mechanism behind leaf color development in Disanthus cercidifolius var. longipes
Source: Front Mol Biosci. 2024 Feb 6;11:1343123. doi: 10.3389/fmolb.2024.1343123 (PMC10876866; doi:10.3389/fmolb.2024.1343123)

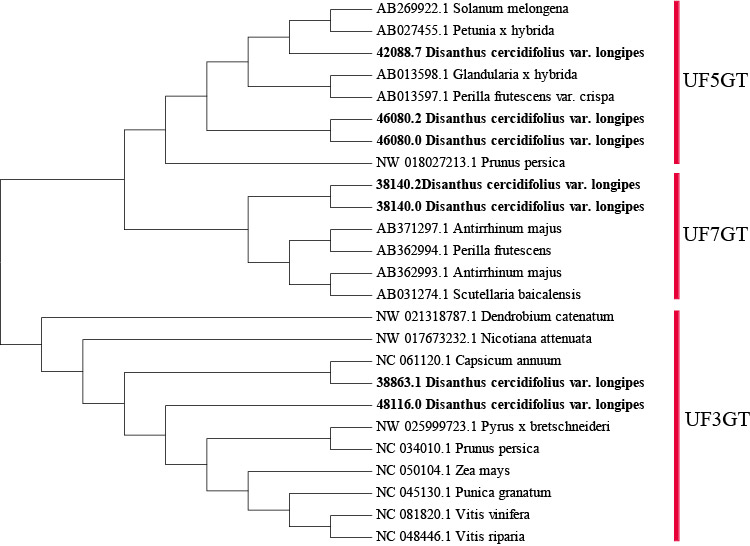

Supplement: Supplementary file 1 [file Image1.JPEG]
