# Supplementary material for: Transcriptomic and metabolic analysis unveils the mechanism behind leaf color development in Disanthus cercidifolius var. longipes
Source: Front Mol Biosci. 2024 Feb 6;11:1343123. doi: 10.3389/fmolb.2024.1343123 (PMC10876866; doi:10.3389/fmolb.2024.1343123)
Supplement: Supplementary file 2 [file Table1.DOC]

| **Supplement Table 1**. qRT-PCR primers used in this study. | | |  |
| --- | --- | --- | --- |
| Gene ID | Direction | Primer sequence (5′–3′) |  |
| Cluster-46055.1 | Forward | TCCACACACATCCATCATCTCTTG | |
|  | Reverse | GTTTGGGTTGAATTTTAGCTACAG | |
| Cluster-26458.4 | Forward | TGTGGGTGCTGCTGTGGGAATG | |
|  | Reverse | TGGGAATCGACGGTGGACAAGG | |
| Cluster-22357.0 | Forward | ACAGCCAATAGCCCAGAACCAA | |
|  | Reverse | CGCCAGGATATGAGCAGAGGAT | |
| Cluster-33493.0 | Forward | CTGGACCTCAACTTCTCGAACC | |
|  | Reverse | CTGGCACCACAACTTGCTCATT | |
| Cluster-46630.1 | Forward | GGGCTCACTTATCTCCTTGTCC | |
|  | Reverse | CACGCAGTAGATTGAGTTGGTC | |
| Cluster-42088.7 | Forward | TACCCTGAATACAACATCCCTACA | |
|  | Reverse | CTAAACTATACTTGAACTGGAACCC | |
| Cluster-47221.1 | Forward | ACGGCACAACAAATGTAACGACC | |
|  | Reverse | TCCAAGACACTAGCTGAGAAGGC | |
| Cluster-38863.1 | Forward | GAAGAAGGGGTGGTAGAGAGAGA | |
|  | Reverse | AGTAGCAACAGCGGTAGCAGAAA | |
| Cluster-38140.2 | Forward | TTCCTCACCTTTCCTCCAACTTTTC | |
|  | Reverse | TATTCTAACTAACCTAATCCGGCCT | |
| Cluster-46444.5 | Forward | ATCCATCAAAAAACCGTGAAAAAA | |
|  | Reverse | CATACCCTCTCCGTGAAAGCAAAT | |
